# Caring for critically ill patients outside intensive care units due to full units: a cohort study

**DOI:** 10.6061/clinics/2017(09)08

**Published:** 2017-09

**Authors:** Fabiane Urizzi, Marcos T. Tanita, Josiane Festti, Lucienne T.Q. Cardoso, Tiemi Matsuo, Cintia M.C. Grion

**Affiliations:** I Pos-graduacao, Hospital Universitario, Universidade Estadual de Londrina, Londrina, PR, BR; II Unidade de Terapia Intensiva Adulto, Hospital Universitario, Universidade Estadual de Londrina, Londrina, PR, BR; III Departamento de Medicina Interna, Hospital Universitario, Universidade Estadual de Londrina, Londrina, PR, BR; IV Departamento de Estatistica, Universidade Estadual de Londrina, Londrina, PR, BR

**Keywords:** Intensive Care Units, Bed Occupancy, Health Services Accessibility, Direct Service Costs, Costs and Cost Analysis

## Abstract

**OBJECTIVES:**

This study sought to analyze the clinical and epidemiologic characteristics of critically ill patients who were denied intensive care unit admission due to the unavailability of beds and to estimate the direct costs of treatment.

**METHODS:**

A prospective cohort study was performed with critically ill patients treated in a university hospital. All consecutive patients denied intensive care unit beds due to a full unit from February 2012 to February 2013 were included. The data collected included clinical data, calculation of costs, prognostic scores, and outcomes. The patients were followed for data collection until intensive care unit admission or cancellation of the request for the intensive care unit bed. Vital status at hospital discharge was noted, and patients were classified as survivors or non-survivors considering this endpoint.

**RESULTS:**

Four hundred and fifty-four patients were analyzed. Patients were predominantly male (54.6%), and the median age was 62 (interquartile range (ITQ): 47 - 73) years. The median APACHE II score was 22.5 (ITQ: 16 - 29). Invasive mechanical ventilation was used in 298 patients (65.6%), and vasoactive drugs were used in 44.9% of patients. The median time of follow-up was 3 days (ITQ: 2 - 6); after this time, 204 patients were admitted to the intensive care unit and 250 had the intensive care unit bed request canceled. The median total cost per patient was US$ 5,945.98.

**CONCLUSIONS:**

Patients presented a high severity in terms of disease scores, had multiple organ dysfunction and needed multiple invasive therapeutic interventions. The study patients received intensive care with specialized consultation during their stay in the hospital wards and presented high costs of treatment.

## INTRODUCTION

The intensive care unit (ICU) provides a continuous monitoring system for critically ill patients who have the potential for recovery or are in a life-threatening situation. In recent decades, this sector has observed a significant increase in the demand for beds associated with the reduced mortality among patients admitted. Additionally, an increase in the complexity of diseases and number of chronic health conditions has been reported 1. A patient who may not benefit from treatment due to either a very good or very poor prognosis may be rejected from the ICU. Some patients, however, may be denied admission to the ICU due to a lack of available beds 2, and this delay in admission has been associated with increased mortality 3.

The concept of rapid and early care has been established in various fields of medicine 4-6. Considering that most acute illnesses develop in stages of physiological and organ dysfunction, the logical step would certainly be to provide specialized care for any critically ill patient within the hospital, regardless of the location. This approach has been described as an “intensive care system without walls” 7.

In an attempt to provide intensive care outside the ICU environment, rapid response teams (RRTs) have been created with the goal of early identification of the signs and symptoms of physiological worsening in patients, thereby reducing the risk of adverse events in inpatient units. This strategy consists of a bedside intensive therapy system formed usually by a doctor, nurse and physiotherapist 8. The benefits that this system have provided to hospitalized patients have been described by several authors 9-15. Most studies have adopted “before and after” quasi-experimental designs, demonstrating a reduction in adverse events in hospitalized patients and hospital mortality. However, there are few studies describing the care costs of critically ill patients outside of the intensive care environment. Research in this area is important for the financial planning of actions in health care.

The aim of the present study was to analyze the clinical and epidemiological characteristics of critically ill patients who were denied ICU admission due to the unavailability of beds and to estimate the direct costs of treatment during this period.

## METHODS

This was a prospective cohort study of critically ill patients who were treated outside the ICU due to a full unit and attended by the RRT in a university hospital from February 2012 to February 2013.

The study was conducted in adult medical-surgical admission wards at the University Hospital of the Londrina State University (HU/UEL). The HU/UEL is a supplementary service of the State University of Londrina and is characterized as a university public hospital, with 330 beds, including 20 adult ICU beds. The RRT in this hospital is composed of an intensivist physician and a physiotherapist who are assigned exclusively to the tasks of this team. The RRT responds to yellow and blue codes, assists in the care of all critically ill patients denied ICU beds and evaluates patients post-discharge from the ICU to prevent early readmissions. For a yellow or blue code, the nurse caring for the patient on the ward triggers a call for RRT consultation and participates in the care of the patient together with the two members of the RRT. During this evaluation, if there is a need to transfer the patient to the ICU, the RRT accompanies this intra-hospital transportation. Patients denied ICU beds remain under the care of the local staff with the support of the RRT. In these cases, the RRT performs two scheduled daily evaluations of these patients to assist with medical prescriptions, clinical decisions, therapeutic interventions, and checking of laboratory and other exam results. If additional evaluations are needed, the local staff calls the RRT.

Triage decisions when there is a lack of a sufficient number of ICU beds are made according to the Society of Critical Care Medicine’s guidelines 16. The ICU request is performed in the electronic hospital system that provides the diagnostic and clinical data of the patient. If there is no ICU bed immediately available, the intensivist on duty with the RRT evaluates the request and classifies the patient according to the prioritization model 16. If there is more than one patient in the first level of prioritization and only one bed is available, then the length of time waiting for the ICU bed is taken into consideration, and the “first come, first served” rule is applied. All adult ICU beds are made available for ICU requests in the electronic system.

A convenience sample was obtained from all adult patients admitted in the study period who presented with a critical condition requiring admission to a monitored ICU bed and who were refused admission due to lack of availability. Patients under the age of 18 years and those who had a waiting time for admission to the ICU of less than 24 hours were excluded.

The data collected consisted of demographic data (age and sex) and clinical data, including the diagnosis of a critical condition, presence of comorbidities, length of stay before ICU admission, data for the calculation of costs and prognostic scores and outcomes. The source of the data was the patient’s records, which were consulted during their stay in the hospital, and the data were then transcribed using instruments designed to carry out the present research.

Each patient was followed until one of the following primary outcomes occurred: ICU admission, cancellation of the request for the ICU due to clinical improvement or limitation of therapeutic support, transfer to another hospital or death. Data collection began on the day of refusal of admission to the ICU and continued until a primary outcome occurred. Patients were classified as survivors and non-survivors considering their vital status at hospital discharge.

The Acute Physiology and Chronic Health Evaluation (APACHE II), Sequential Organ Failure Assessment (SOFA) and Therapeutic Intervention Scoring System (TISS-28) scores were collected. The APACHE II score was calculated to characterize the severity of the study population. The SOFA score was used to observe variations in organ dysfunction/failure. The TISS-28 score was used to quantify therapeutic interventions carried out during treatment outside the ICU. Each of these scores was calculated according to their original descriptions 17-19.

In an interim analysis, a smaller sample of patients estimated to be representative was used to perform a cost analysis. For this purpose, data were collected from patients included in the study period from February to July 2012. The model adopted to collect costs applied the "bottom-up" approach, a methodology considered the gold standard, in which the goal is to estimate costs by individual patient or by a group of patients 20. The measurement of direct costs is then generated, and the sum of the costs can provide a conservative estimate of the true value of treating a health problem 21. For the present study, the direct costs generated for the treatment of patients were divided into four categories: clinical support, consumer items, human resources and hospital fees.

1. Clinical support: costs related to pharmacy needs (ointments, body oil, strips for verification of blood sugar and items for pressure ulcer prevention), renal support, laboratory, laboratory tests, imaging and complementary examinations.
2. Consumer items: costs of medication, nutrition, blood and its derivatives.
3. Human resources: medical procedures and physical therapists.
4. Hospital fees: considered a fixed daily rate, independent of the type of disease.

Items from the clinical support category were analyzed according to the medical prescription. For the calculation of medication costs, a standard dose was considered, calculated as the mean daily prescription for a patient of 70 kg body weight. In the category of human resources, values were attributed to medical procedures performed as well as the physiotherapy service, which was divided into motor and respiratory therapy, as noted in the medical record. In the category of hospital fees, the values of the daily hospital fees and intensivist doctor on duty with the RRT were computed. Costs related to the use of equipment, infrastructure, electricity, security systems, information technology, and non-clinical support and indirect costs (lost productivity, etc.) were not analyzed.

After data collection, a value was assigned to all items. The values were obtained from standard tables and index values for medical procedures outlined by the Brazilian Medical Association (BMA) 22. For items of hospital consumption, medications and solutions, the Brasíndice price list 23 version 799, year 2014, was used. Subsequently, the values were translated into US dollars (US$) based on the average price of currency for the year 2013.

Comorbidities were defined according to the criteria published in the Charlson comorbidity index 24. The need for ICU admission was classified as one of the following: respiratory failure; hemodynamic instability; metabolic disorder, postoperative; cardiac monitoring; neurological monitoring; or other. The diagnosis of infection was based on clinical, microbiological and imaging results, and the source of infection was classified as lung, urinary tract, bloodstream, abdominal, surgical site or other. The diagnosis and classification of sepsis used the Third International Consensus Definitions for sepsis and septic shock 25. Sepsis was defined as life-threatening organ dysfunction caused by a dysregulated host response to infection, and septic shock was defined as a subset of sepsis in which particularly profound circulatory, cellular, and metabolic abnormalities were associated with a greater risk of mortality than with sepsis alone.

### Statistical analysis

Data were analyzed using SPSS version 19.0 (Armonk, NY: IBM Corp.) and MedCalc version 15 (MedCalc Software, Mariakerke, Belgium); the significance level was 5%, and the confidence interval was 95%. Continuous quantitative variables were described after the normality of the distribution was verified with the Shapiro-Wilk test. For variables that presented a normal distribution, the mean and standard deviation were calculated; otherwise, the median and interquartile range (ITQ) (25^th^ percentile - 75^th^ percentile) were calculated. The nominal categorical variables were described as absolute and relative frequencies (%) of each variable. Categorical variables were compared using Pearson’s chi-square test (χ^2^) or Fisher’s exact test, where over 20% of the expected frequencies in the tables were lower than five. Continuous variables were compared using Student’s t-test or the Mann-Whitney test according to the data distribution. The area under the receiver operating characteristic (ROC) curve was calculated to evaluate the accuracy and compare the performance of the APACHE II, SOFA and TISS-28 to discriminate survivors and non-survivors. The areas under the ROC curve of the indices were compared in pairs using a non-parametric approach, based on the difference between the areas and standard error.

### Ethical approval and consent to participate

This study was approved by the Ethics Committee for Research Involving Human Beings, State University of Londrina/Northern Paraná Regional University Hospital, as Opinion No. 281/2010, December 17, 2010, CAAE No. 0255.0.268.000-10. The ethics committee waived the need for informed consent.

## RESULTS

During the study period, 675 critically ill patients had ICU bed requests denied and were cared for in the hospital wards with intensivist consultation by the RRT. There were 221 patients excluded as follows: 60 patients were under 18 years of age, 102 patients spent less than 24 hours waiting for the ICU bed, and 59 patients were considered losses due to a lack of sufficient information in the medical records to complete the case report form. In total, 454 patients were evaluated. Among the patients studied, data collection on direct costs was performed for 151 during the period from February to July 2012.

Of the 454 patients included in the study, 54.6% were male, and the median age was 62 (47-73) years. The median APACHE II severity score was 22.5 16-29. Organ dysfunction measured by the SOFA score at study entry presented a median of 8 4-10, and the SOFA score at the time of primary outcome was 8 4-13. At this point, 159 patients (35%) presented a SOFA outcome lower than the SOFA score at study entry; for 53 patients (11.7%), this score did not change during the analysis, and for 242 patients (53.3%), the SOFA outcome score was higher in relation to study entry. The median score for therapeutic interventions (TISS-28) at study entry was 27 21-30.

Regarding the sector of origin, 65% of patients were cared for in the emergency room, 34.8% in the general wards and 0.2% in the operating room. According to the diagnostic categories of the APACHE II system, 92.3% of patients were considered clinical, and 7.7% were surgical. For the diagnosis at the time of requesting an ICU bed, according to the standardization of the APACHE II system, sepsis was the most frequent diagnosis (62.2%), followed by the principal "cardiovascular" system (12.8%), the principal "neurological" system (10%), postoperative conditions (3.2%) and other diagnoses (11.8%).

A diagnosis of infection was present in 366 (80.6%) patients at some time during the study period, and the identified sources of infection were the lungs (76.5%), urinary system (9%), abdominal area (6%), skin and soft tissue (5.2%), and the bloodstream, surgical site or other source (3.3%). Regarding the classification of infections, 198 patients presented septic shock, 158 had sepsis, and 10 had a localized infection.

In 39% of cases, the reason for ICU admission was respiratory failure, followed by hemodynamic instability (36.3%), neurological monitoring (14.5%), cardiac monitoring (7.0%) and postoperative care (2.9%). The most frequent comorbidities were hypertension (19.3%), diabetes mellitus (18.5%), congestive heart failure (14.5%), cirrhosis (8.4%), chronic renal failure (6.6%), immunosuppression (6.1%) and other (26.6%).

Mechanical ventilation was required in 298 patients (65.6%) at some point while they waited for an ICU vacancy, and of the 156 patients who were on spontaneous ventilation, 91 required respiratory support with oxygen therapy. When mechanical ventilation was initiated, an intensivist physiotherapist was assigned to care for the patient and consult in cases of any difficulties regarding this intervention. The need for therapeutic interventions was associated with higher mortality (Table 1).

In relation to the treatment of hemodynamic instability, 44.9% of patients used vasopressors or inotropic agents, including norepinephrine (36.1%), adrenaline (4.2%), dobutamine (3.1%) and dopamine (1.5%). On the other hand, 5.9% of patients required the use of vasodilators, including sodium nitroprusside (3.3%) and nitroglycerin (2.6%), and 14.9% of patients required the use of multiple vasoactive drugs at some point during the study period.

Of the therapeutic interventions in these patients, 51 patients required procedures that are usually performed in an ICU but needed to be carried out in the hospital wards, such as tracheal intubation, insertion of a transvenous pacemaker, hemodialysis, and insertion of a chest tube. In addition, 150 patients required intra- or inter-hospital transportation for the performance of diagnostic or therapeutic procedures.

Of the 454 patients analyzed, 204 (44.9%) were admitted to the ICU after a waiting period of 3 days 2-6. Two patients (0.4%) were transferred to another hospital, and 25 patients (5.5%) had therapeutic support limited after agreement by the head doctor and family because they presented an irreversible clinical condition and therapeutic interventions were considered futile. While waiting for an ICU bed, 101 patients (22.3%) died and 122 patients (26.9%) had their ICU admission requests canceled due to clinical improvement.

The demographic characteristics and prognostic scores of the group of patients whose data were collected for the calculation of direct costs were no different from those of the other patients in the study. Patients not included in the cost analysis stayed longer in the hospital, but the period of cost analysis was similar. Patients included in cost analysis also required vasoactive drugs more frequently (Table 2).

**Figure 1:**
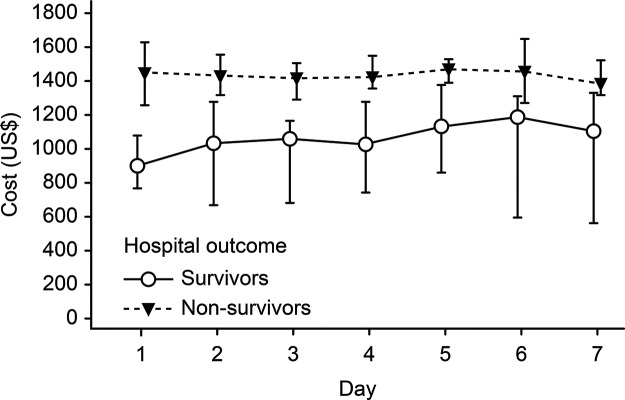
Median daily costs and 95% confidence intervals for the survivors and non-survivors in the first week of the study period.

The median total cost per patient was US$ 5,945.98 (US$ 3,831.98 - US$ 10,073.41), while the median daily cost was US$ 1,618.51 (US$ 1,235.29 - US$ 2,032.40). Considering the blocks of direct costs, the median total cost of laboratory tests was US$ 502.82 (US$ 255.61 - US$ 778.37); medical procedures, US$ 81.81 (US$ 0.00 - US$ 525.28); medications, US$ 4,342.51 (US$ 2,452.17 - US$ 7,713.01); and clinical support, US$ 298.71 (US$ 45.64 - US$ 551.78). In addition, the costs in accordance with outcomes were analyzed (survivors and non-survivors), and there was a higher resource consumption of medicines in patients who died (US$ 4,532.12 [US$ 2,698.91 - US$ 9,022.49]) than among those who were discharged alive (US$ 3,594.37 [US$ 1,231.11 - US$ 6,136.93]), *p*=0.009. The same finding was observed in relation to the total costs, with a median of US$ 6,159.26 (US$ 4,038.63 - US$ 11,257.26) in non-survivors and US$ 5,015.29 (US$ 2,845.04 - US$ 8,207.94) in survivors, *p*=0.041 (Table 3). To compare the day-by-day costs between survivors and non-survivors, the results for the first seven days of observation are presented in Figure 1. The results showed that the median daily cost was higher in non-survivors.

## DISCUSSION

The present study describes the care of critically ill patients outside the ICU in the hospital wards, which is becoming a common reality for hospitals around the world. Caring for these patients with daily intensivist consultations and the aid of an RRT was a local solution to increase safety for these hospitalized patients. These patients required mechanical ventilation, vasoactive drugs and invasive procedures, and they received such assistance outside of a monitored ICU bed. This situation is associated with high costs of care and possibly with an increase in adverse events.

There are few studies in the literature evaluating the level of care and outcomes of critically ill patients treated outside the ICU. Previous studies 26,27 have described the benefits of transfer to an ICU bed in a period of up to three days and have shown that patients with an APACHE II score above 16 benefit the most from early admission (up to 3 days) to an ICU 27. Usually, the critically ill patient is identified and treated by the RRT and promptly transferred to an ICU to receive continued care. However, it is also possible that the RRT continues to provide critical care for patients who are denied ICU admission and remain in the hospital wards awaiting ICU bed availability. Unfortunately, this situation is becoming increasingly frequent, particularly in low- and middle-income countries. Despite the attempt to provide intensive care for patients outside the ICU in this study institution, a small proportion of the patients became refractory to treatment after hours or days of waiting for an ICU bed. It is possible that these patients’ clinical conditions became refractory due to sub-optimal treatment provided outside the ICU, and it is conceivable that patients may die due to the lack of ICU beds 3.

Since most critical illnesses are time sensitive, delaying ICU admission can lead to a worse prognosis if patients do not have access to adequate care, but there are conflicting data in the literature regarding this issue. Some authors describe a 1.5% increase in the risk of death for each hour of delay in ICU admission 3 for patients treated in hospital wards, while others have described no change in prognosis for surgical patients cared for in the post-anesthesia care unit 28.

The direct costs associated with the care of these patients were high and comparable with the costs described in the literature regarding ICU patients 26,27,29-32. In the present study, the cost of non-surviving patients was higher than that of survivors. Jacobs et al., in an analysis of the daily cost of ICU patients in the UK, found that the cost of non-survivors was higher than that of survivors, with a daily consumption of a mean of £578.00 for all patients and £748.00 for non-survivors 29. Other factors that determined costs included mechanical ventilation and the APACHE II score. It was also noted in France that the cost of non-survivors was higher than that of survivors, with a daily average of €1,380.00 for all patients 30. When analyzing diagnostic groups in a multicenter study in Italian ICUs, the authors found that patients with multiple trauma (€4,717.00), acute abdominal issues (€3,529.00) and pneumonia or acute respiratory distress syndrome (€3,946.00) presented the highest levels of resource consumption 31.

In the present study, it was observed that 80.6% of the diagnoses were related to infection and its complications. The first and largest multicenter study conducted in Brazil on this issue noted that the cost of treatment of septic patients was high, and the median daily cost was higher in non-survivors (US$ 1,094.00) compared to survivors (US$ 826.00) 32. In an evaluation of the economic impact of implementing a hospital protocol for sepsis, Koening et al. also noted that the mean overall costs were higher for non-survivors (US$ 27,308.00) than for survivors (US$ 20,021.00) 33.

In other countries, such as the United States 34, Italy 35, Germany 36 and France 37, it has also been shown that resource consumption was higher in non-surviving septic patients than among survivors. Regarding this point, it is worth noting that each country is unique in relation to its health system, management models, reimbursement rates and cost components; therefore, such comparisons should be interpreted with caution. However, this finding occurred independent of national differences and characteristics.

The findings of the present study contribute to the planning of resource allocation, admission protocols with a prioritization model, investment in staff, and training courses.

There are some limitations to the present study that should be considered. The first is related to the analysis of a single center; thus, interpretation of the results must be performed with caution and be limited to institutions with similar characteristics. The second refers to the observational design, which may be prone to selection bias and could have affected the results.

This study is novel in its analysis of the costs of critically ill patients treated outside the ICU, and this contribution is the main strength of this study. Another strength of this study was the rigor used in the cost analysis, which applied the methodology considered the "gold standard". Moreover, the results are relevant, comparable and reproducible for other institutions with the same characteristics.

Critically ill patients treated outside the ICU presented prognostic scores that demonstrated a high degree of organ dysfunction and required a large number of therapeutic interventions, including vasoactive drugs and invasive mechanical ventilation. The direct costs of treatment of these critically ill patients were high and associated with a poor prognosis.

## AUTHOR CONTRIBUTIONS

Urizzi F, Matsuo T and Grion CM participated in the study concept and design. Urizzi F, Tanita MT, Festti J and Cardoso LT carried out the acquisition of data and participated in the analysis and interpretation of data. Urizzi F and Grion CM drafted the manuscript. Matsuo T performed the statistical analysis. All authors participated in the critical revision of the manuscript for intellectual content and read and approved the final version of the manuscript.

## Figures and Tables

**Table 1:** Demographic and clinical characteristics of the study patients.

	All	Survivors (n=154)	Non-survivors (n=300)	P value
Age (years)	62 (47-73)	56.5 (37-67)	64 (49.5-75)	<0.001*
Males (%)	248 (54.6)	69 (44.8)	179 (59.6)	0.002†
Time before ICU (days)‡	3 (2-6)	2 (2-4)	2 (2-3)	0.43*
Length of hospital stay (days)	16 (7-31)	14 (8-28)	2 (2-3)	<0.001*
ICU indication				
Clinical (%)	419 (92.3)	136 (88.3)	283 (94.3)	0.023†
Surgery (%)	35 (7.7)	18 (11.7)	17 (5.7)	
Reason for ICU admission				
Respiratory failure	177 (39)	43 (27.9)	134 (44.7)	<0.001†
Hemodynamic instability	165 (36.3)	46 (29.9)	119 (39.7)	
Neurological monitoring	66 (14.5)	30 (19.5)	36 (12)	
Cardiac monitoring	32 (7)	27 (17.5)	5 (1.7)	
Postoperative care	13 (2.9)	7 (4.5)	6 (2)	
Other	1 (0.2)	1 (0.6)	0 (0)	
APACHE II	22.5 (16-29)	16 (9-21)	25 (20-31)	<0.001*
SOFA	8 (4-10)	4 (2-7)	9 (6-11)	<0.001*
TISS-28	27 (21-30)	22 (16-28)	29 (24-32)	<0.001*
VM (%)	298 (65.6)	69 (23.2)	229 (76.8)	<0.001†
Vasoactive drugs (%)	204 (44.9)	35 (17.2)	169 (82.8)	<0.001†

Legend: ICU - intensive care unit; APACHE II - Acute Physiology and Chronic Health Evaluation; SOFA - Sequential Organ Failure Assessment; TISS-28 - Therapeutic Intervention Scoring System - 28; VM - mechanical ventilation.

* Mann-Whitney test;

† Fisher's exact test;

‡ Period between ICU request and admission or cancellation.

**Table 2:** Comparison of demographic and clinical characteristics of the study patients who were or were not included in the analysis of direct costs.

	In cost analysis (n=151)	Not in cost analysis (n=304)	P value
Age (years)*	64 (49-72)	60 (43-75)	0.32†
Males (%)	83 (55.0)	165 (54.5)	0.92‡
Time before ICU (days)§	3 (2-6)	4 (2-6)	0.32†
Length of hospital stay (days)	21 (11-38.75)	14 (7-28)	<0.001†
ICU indication			
Clinical (%)	140 (92.7)	279 (92.1)	0.81‡
Surgery (%)	11 (7.3)	24 (7.9)	
Reason for ICU admission			
Respiratory failure	67 (44.4)	110 (36.3)	0.40‡
Hemodynamic instability	49 (32.5)	116 (38.3)	
Neurological monitoring	23 (15.2)	43 (14.2)	
Cardiac monitoring	10 (6.6)	22 (7.3)	
Postoperative care	2 (1.3)	11 (3.6)	
Other	0 (0)	1 (0.3)	
APACHE II*	23 (16-29)	22 (15-28)	0.11†
SOFA (day 1)*	8 (4-11)	7 (4-10)	0.16†
TISS (day 1)*	27 (21-30)	27 (21-30)	0.43†
VM (%)	106 (70.2)	192 (63.4)	0.15‡
Vasoactive drugs (%)	78 (51.7)	126 (41.6)	0.04‡

Legend:

* Median (interquartile range);

† Mann-Whitney test;

‡ Chi-square;

§ Period between ICU request and admission or cancellation (period of cost analysis)

SOFA (day 1): Sequential Organ Failure Assessment at study entry

APACHE II: Acute Physiology and Chronic Health Evaluation

TISS (day 1): Therapeutic Intervention Scoring System at study entry.

**Table 3:** Median cost (US$) of critically ill patients treated outside intensive care units according to the hospital outcome.

Variable	Total (n=151)	Survivors (n=53)	Non-survivors (n=98)	P value*
Laboratory tests	502.82 (255.61-778.37)	505.54 (241.74-753.81)	499.52 (272.00-821.14)	0.685
Medical procedures	81.81 (0.00-525.38)	9.26 (0.00-598.86)	81.81 (0.00-528.26)	0.551
Medications	4342.51 (2452.17-7713.01)	3594.37 (1231.11-6136.93)	4532.12 (2698.91-9022.49)	0.009
Clinical support	298.71 (45.65-551.78)	275.89 (45.65-565.69)	315.13 (39.70-549.67)	0.881
Total cost	5945.98 (3831.98-10073.41)	5015.29 (2845.04-8207.94)	6159.26 (4038.63-11257.26)	0.041
Daily cost	1618.51 (1235.29-2032.40)	1269.71 (847.76-1653.87)	1787.36 (1441.40-2280.60)	<0.001

Median data (interquartile deviation)

* Mann-Whitney test.
